# Lower Respiratory Tract Infection Induced by a Genetically Modified Picornavirus in Its Natural Murine Host

**DOI:** 10.1371/journal.pone.0032061

**Published:** 2012-02-15

**Authors:** Louis A. Rosenthal, Renee J. Szakaly, Svetlana P. Amineva, Yina Xing, Marchel R. Hill, Ann C. Palmenberg, James E. Gern, Ronald L. Sorkness

**Affiliations:** 1 Department of Medicine, University of Wisconsin School of Medicine and Public Health, Madison, Wisconsin, United States of America; 2 Department of Pediatrics, University of Wisconsin School of Medicine and Public Health, Madison, Wisconsin, United States of America; 3 Institute for Molecular Virology, University of Wisconsin-Madison, Madison, Wisconsin, United States of America; 4 School of Pharmacy, University of Wisconsin-Madison, Madison, Wisconsin, United States of America; Instituto Butantan, Brazil

## Abstract

Infections with the picornavirus, human rhinovirus (HRV), are a major cause of wheezing illnesses and asthma exacerbations. In developing a murine model of picornaviral airway infection, we noted the absence of murine rhinoviruses and that mice are not natural hosts for HRV. The picornavirus, mengovirus, induces lethal systemic infections in its natural murine hosts, but small genetic differences can profoundly affect picornaviral tropism and virulence. We demonstrate that inhalation of a genetically attenuated mengovirus, vMC_0_, induces lower respiratory tract infections in mice. After intranasal vMC_0_ inoculation, lung viral titers increased, peaking at 24 h postinoculation with viral shedding persisting for 5 days, whereas HRV-A01a lung viral titers decreased and were undetectable 24 h after intranasal inoculation. Inhalation of vMC_0_, but not vehicle or UV-inactivated vMC_0_, induced an acute respiratory illness, with body weight loss and lower airway inflammation, characterized by increased numbers of airway neutrophils and lymphocytes and elevated pulmonary expression of neutrophil chemoattractant CXCR2 ligands (CXCL1, CXCL2, CXCL5) and interleukin-17A. Mice inoculated with vMC_0_, compared with those inoculated with vehicle or UV-inactivated vMC_0_, exhibited increased pulmonary expression of interferon (IFN-α, IFN-β, IFN-λ), viral RNA sensors [toll-like receptor (TLR)3, TLR7, nucleotide-binding oligomerization domain containing 2 (NOD2)], and chemokines associated with HRV infection in humans (CXCL10, CCL2). Inhalation of vMC_0_, but not vehicle or UV-inactivated vMC_0_, was accompanied by increased airway fluid myeloperoxidase levels, an indicator of neutrophil activation, increased MUC5B gene expression, and lung edema, a sign of infection-related lung injury. Consistent with experimental HRV inoculations of nonallergic, nonasthmatic human subjects, there were no effects on airway hyperresponsiveness after inhalation of vMC_0_ by healthy mice. This novel murine model of picornaviral airway infection and inflammation should be useful for defining mechanisms of HRV pathogenesis in humans.

## Introduction

Infections with the picornavirus, human rhinovirus (HRV), are the most frequent cause of the common cold. However, HRV infections, which usually cause self-limiting upper respiratory tract illnesses, are also the leading cause of virus-induced asthma exacerbations [1], and HRV wheezing illnesses in the first few years of life are associated with increased risk for the development of childhood asthma [2]–[4]. The mechanisms by which a common cold virus can induce asthma exacerbations and contribute to the development of persistent lower airway sequelae in susceptible children remain to be elucidated [5], [6].

There is considerable evidence that HRV can infect the lower respiratory tract [7]–[14] and that HRV infection stimulates the production of proinflammatory chemokines and cytokines by lower airway epithelial cells [15]. Neutrophils are the main first-line inflammatory cells recruited to the airways during HRV infections [16]–[18], and this neutrophilic inflammatory response has been associated with asthma symptoms and airway dysfunction [19]–[22]. However, the relationship between neutrophilic airway inflammation and HRV-induced airway disease is still largely undefined. The eventual outcome of HRV infection, a relatively uneventful upper respiratory tract illness versus a more severe lower respiratory tract illness, might be related to the balance between detrimental and beneficial effects of the neutrophilic inflammatory response in the airways, which might be influenced by host, viral, developmental, and environmental factors. The development of useful small animal models of picornavirus-induced neutrophilic airway inflammation could facilitate mechanistic studies to address these issues.

There are no known murine rhinoviruses, which has significantly hampered the investigation of the mechanisms governing the inflammatory responses to HRV infection and the subsequent development of airway sequelae. Experimental models using either minor receptor group HRV in wild-type mice or major receptor group HRV in mice that are transgenic for human intercellular adhesion molecule-1 (ICAM-1; CD54), the receptor for major group HRV, have been developed recently [23]–[28]. A limitation of these useful models is that HRV titers exhibit a steep decline after inoculation of normal mice [23]–[26]. The development of rodent models in which picornavirus replication persists for several days in the airways of unmanipulated hosts, as in experimental and clinical HRV infections, could facilitate investigation of the relationships between viral replication and the development of airway inflammation and dysfunction. For this reason, we have explored the use of a murine picornavirus, which replicates efficiently in its natural hosts, to model picornavirus-induced respiratory infections.

Mice are the natural hosts for mengovirus, a picornavirus whose wild-type form causes infections that are more similar to systemic poliovirus infections than to HRV-induced airway infections [29]. The mengovirus genome has a poly(C) tract in the distal region of its 5′ untranslated region, which has been shown to be an important virulence determinant that inhibits host type I interferon (IFN) responses [30]–[34]. Investigation of a mengovirus mutant, vMC_0_, in which the poly(C) tract had been deleted showed that vMC_0_ induces robust type I IFN responses and that vMC_0_ inoculation by intracerebral or intraperitoneal routes results in self-limited infections rather than the often lethal, systemic infections induced by wild-type mengovirus [30]–[34]. Wild-type mengovirus efficiently replicates in both epithelial and macrophage lineage cells; however, vMC_0_, like HRV, replicates well in epithelial cells but poorly in macrophage lineage cells [34], [35]. These similarities between vMC_0_ and HRV led us to hypothesize that vMC_0_ could produce a respiratory tract infection in rodents akin to HRV infections in humans. Using rats, were able to demonstrate that inoculation of vMC_0_ by an inhalation route could induce infection of the lower airways and neutrophilic airway inflammation [36]. An effective HRV infection model has not been established in rats. Therefore, vMC_0_ should be useful for investigating picornavirus-induced airway infections in rats, and studies to explore the effects of vMC_0_ infection in an established rat model of allergic airway inflammation are underway. However, the much wider range of mutant strains and genetic tools available in the mouse also made it highly desirable to develop a robust murine model of vMC_0_-induced airway infection. In this paper, we demonstrate that inhalation of the genetically attenuated mengovirus, vMC_0_, induces an acute lower respiratory tract illness in mice, which is characterized by replication and persistent shedding of virus and neutrophilic airway inflammation with evidence of neutrophil activation and lung injury. This novel murine model of picornavirus-induced lower respiratory tract infection and inflammation should be useful for investigating mechanisms of HRV pathogenesis in humans.

## Results

### Viral replication and persistent viral shedding in the lungs after inhalation of attenuated mengovirus, vMC_0_

**Figure 1:**
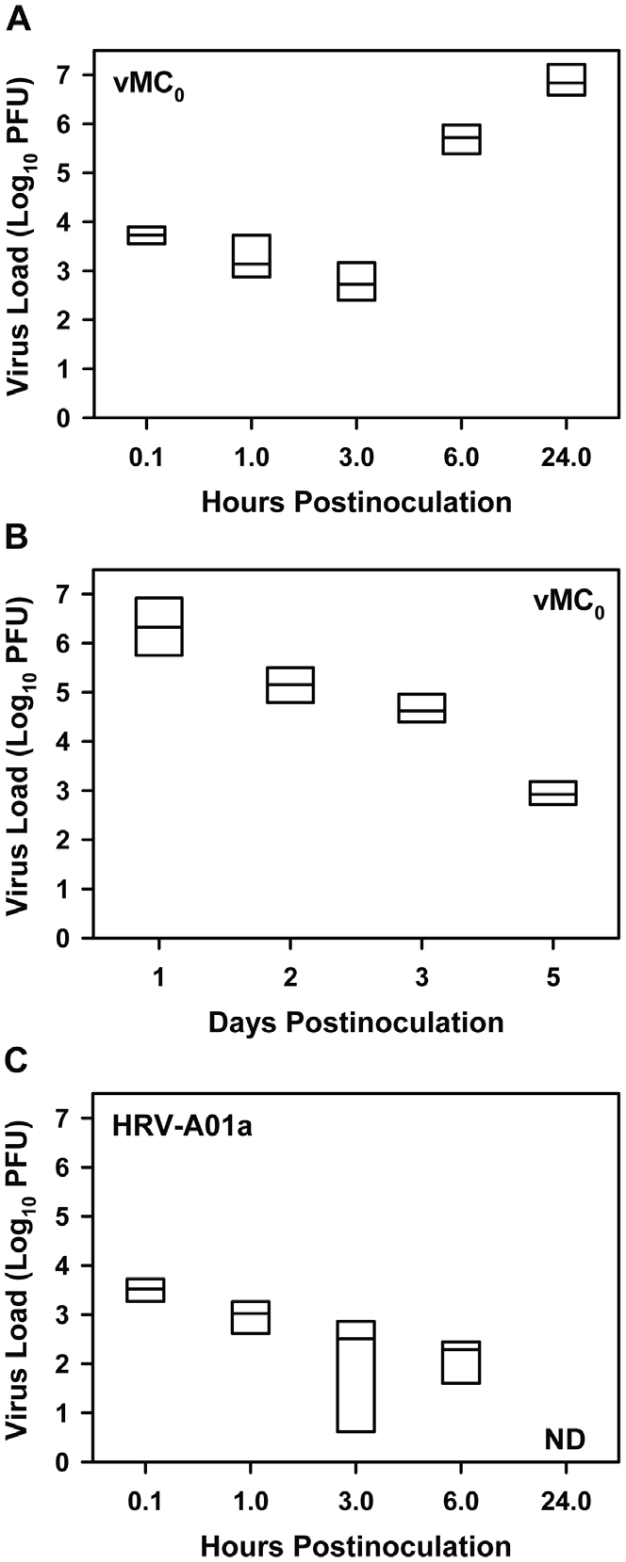
Differences between attenuated mengovirus and HRV in the kinetics of lung viral titers. Mice received intranasal inoculations of 10^6^ PFU of attenuated mengovirus, vMC_0_ [A (n = 7 mice per group), B (n = 6 mice per group)], or 5×10^6^ PFU of HRV-A01a (C; n = 4 mice per group). Lungs were harvested at the indicated times, and viral titers in lung homogenates were determined by plaque assays. Data are the total amount of virus present in the lung homogenates (virus concentrations were multiplied by lung homogenate volumes). No virus was detected in lungs from vehicle-inoculated mice. Data are presented as box plots. For one HRV-A01a-inoculated mouse at 3 h postinoculation, a value of 1 PFU was assigned for graphing purposes because virus was undetectable. ND, not detectable.

After an intranasal inoculation of 10^6^ plaque-forming units (PFU) of attenuated mengovirus, vMC_0_, a median of 5.4×10^3^ PFU were detected in whole lung homogenates from mice at 0.1 h postinoculation, with viral titers remaining relatively stable at 1 and 3 h postinoculation (Fig. 1A). At 6 and 24 h postinoculation, there was a marked increase in viral titers to median levels of 5.3×10^5^ and 6.9×10^6^ PFU, respectively, which is indicative of substantial viral replication in the lung (Fig. 1A). Infectious vMC_0_ continued to be shed in the lungs for at least 5 days postinoculation (Fig. 1B). In contrast, after intranasal inoculation of 5×10^6^ PFU of HRV-A01a, a minor receptor group HRV, a median of 3.4×10^3^ PFU were detected in whole lung homogenates at 0.1 h postinoculation, and viral titers declined until they were undetectable at 24 h postinoculation (Fig. 1C). No virus was detected in vehicle-inoculated mice. These data show that inhalation of vMC_0_, compared with that of HRV-A01a, resulted in more robust viral replication and greater persistence of viral shedding in the lungs.

### Type I and III IFN production in the lungs after inhalation of vMC_0_

**Figure 2:**
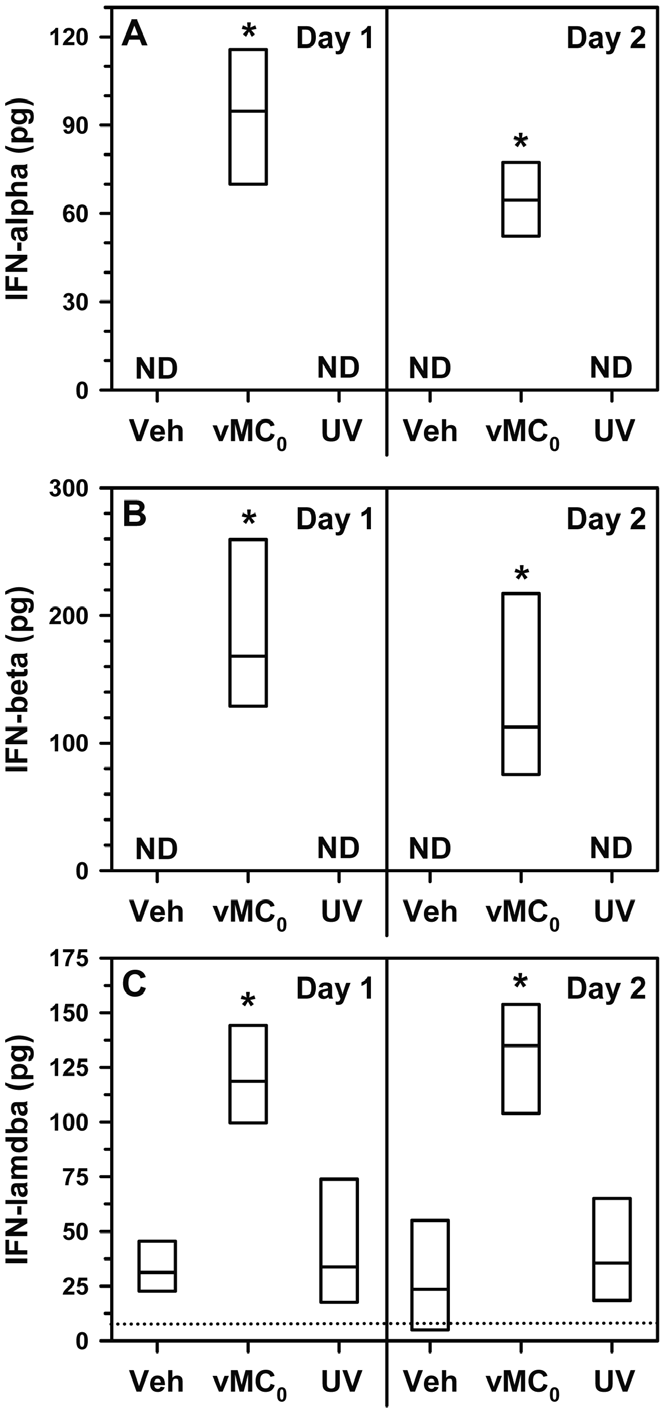
Induction of type I and III IFN expression in response to inhalation of attenuated mengovirus. Mice received intranasal inoculations of 10^6^ PFU of attenuated mengovirus, vMC_0_, an equivalent amount of UV-inactivated vMC_0_, or vehicle (n = 6 mice per group). Levels of (A) IFN-α, (B) IFN-β, and (C) IFN-λ protein in BAL fluid on days 1 and 2 postinoculation were determined by ELISA. Data are the total amount of IFN recovered (ELISA values were multiplied by the BAL fluid volume). IFN-λ protein levels below the limit of detection (dotted line) were assigned a value of 5 pg for graphing purposes. Data are presented as box plots. Veh, vehicle; ND, not detectable. * *P*≤0.01 (vMC_0_ vs. vehicle or UV-inactivated vMC_0_).

The induction of type I and III IFN is a key component of the host response to HRV infection [37]–[39] and an indicator of viral replication. Intranasal inoculation with vMC_0_, but not UV-inactivated vMC_0_, resulted in significant increases in bronchoalveolar lavage (BAL) fluid levels of the type I IFNs, IFN-α and IFN-β, and type III IFN, IFN-λ, compared with those in vehicle-inoculated mice on days 1 and 2 postinoculation (Fig. 2). Thus, vMC_0_-induced IFN production required replication competent virus.

### Pulmonary expression of viral RNA sensors after inhalation of vMC_0_

**Figure 3:**
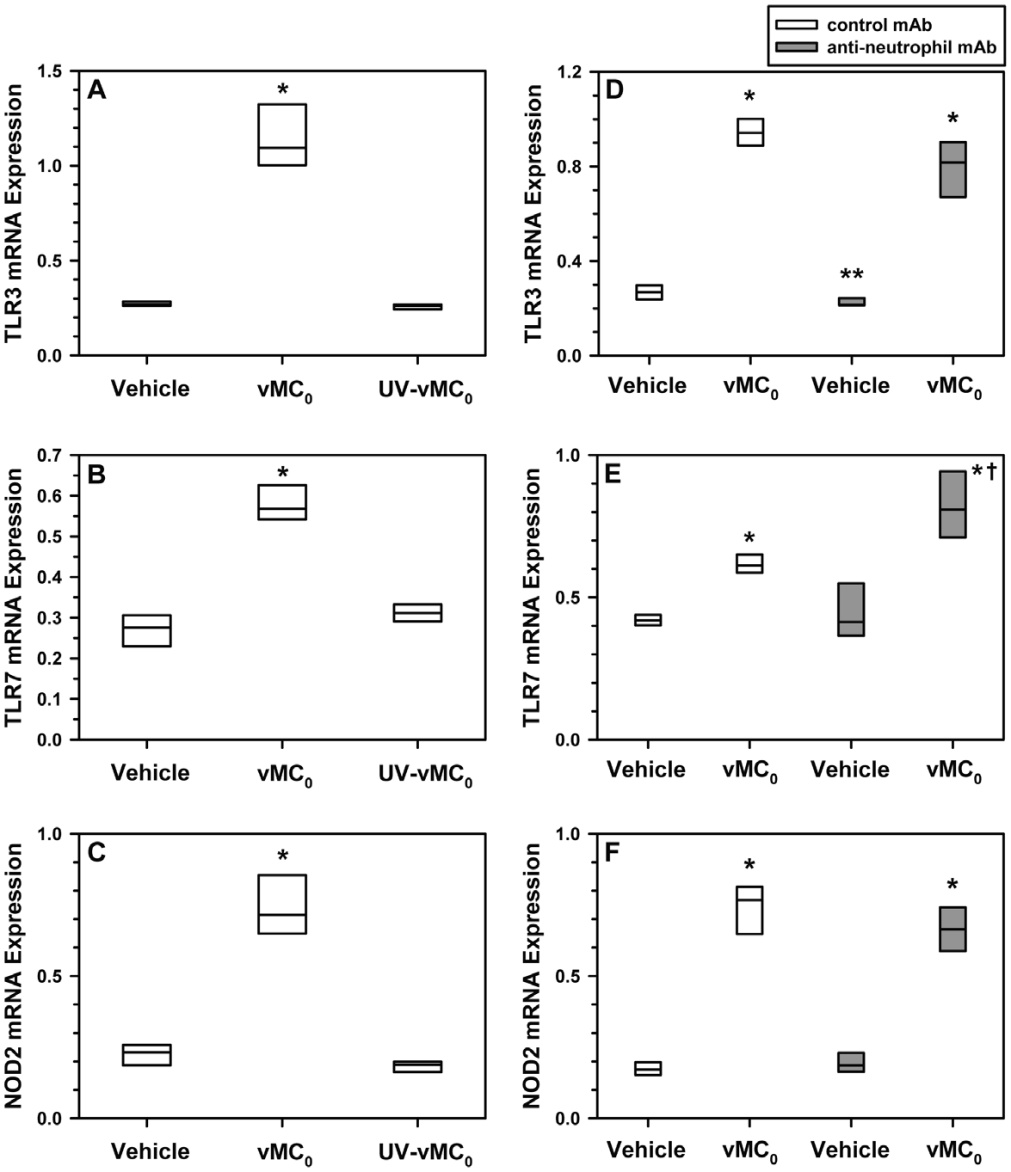
Increased pulmonary expression of viral RNA sensors in response to inhalation of attenuated mengovirus. (A–C) Mice received intranasal inoculations of 10^6^ PFU of attenuated mengovirus, vMC_0_, an equivalent amount of UV-inactivated vMC_0_, or vehicle (n = 6 mice per group). (D–F) Mice were treated with anti-neutrophil mAb or control mAb before receiving intranasal inoculations of 10^6^ PFU of vMC_0_ or vehicle (n = 5–6 mice per group). Levels of (A, D) TLR3, (B, E) TLR7, and (C, F) NOD2 mRNA in lungs on day 1 postinoculation were determined by real-time quantitative RT-PCR and normalized to β-actin mRNA levels. * *P*<0.01 (vMC_0_ vs. vehicle or UV-inactivated vMC_0_), ** *P*<0.05 (anti-neutrophil mAb/Vehicle vs. control mAb/Vehicle), † *P* = 0.01 (anti-neutrophil mAb/vMC_0_ vs. control mAb/vMC_0_).

Pattern recognition receptors, toll-like receptor (TLR)3 and TLR7, have been implicated as viral RNA sensors in the host response to HRV infection [40], [41]. NOD2 (nucleotide-binding oligomerization domain containing 2) is a pattern recognition receptor that detects bacterial peptidoglycan, but which has been shown recently to also function as a viral RNA sensor [42]. Inhalation of vMC_0_, but not UV-inactivated vMC_0_, resulted in significant increases in TLR3, TLR7, and NOD2 mRNA levels in the lungs on day 1 postinoculation compared with mRNA levels in vehicle-inoculated mice, indicating the stimulation of viral RNA sensor pathways (Fig. 3A–C). Pretreatment of mice with an anti-neutrophil monoclonal antibody (mAb) did not result in reduced pulmonary expression of TLR3, TLR7, or NOD2 mRNA in vMC_0_-inoculated mice on day 1 postinoculation in comparison with vMC_0_-inoculated mice that had been pretreated with a control mAb (Fig. 3D–F); indeed, a significant increase in TLR7 mRNA expression was observed in the anti-neutrophil mAb-treated group (Fig. 3E). Thus, the observed increases in TLR3, TLR7, and NOD2 mRNA levels after inhalation of vMC_0_ were apparently due, at least in part, to increased expression in resident lung cells rather than being solely due to the recruitment of neutrophils that might express these receptors. In vehicle-inoculated mice, pretreatment with the anti-neutrophil mAb resulted in a significant decrease in TLR3 mRNA levels compared with pretreatment with the control mAb (Fig. 3D).

### Body weight loss after inhalation of vMC_0_

**Figure 4:**
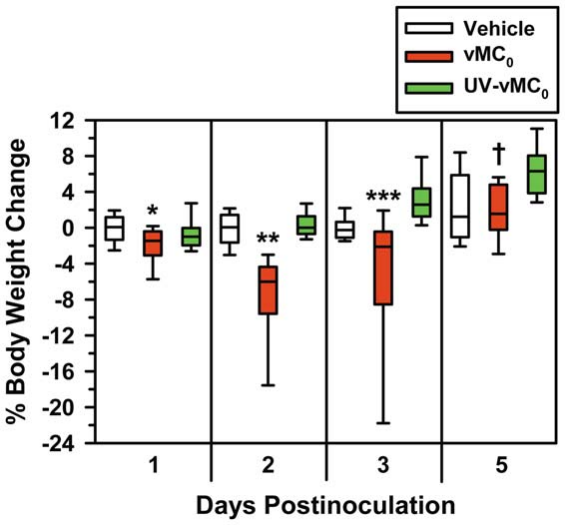
Body weight reduction in response to inhalation of attenuated mengovirus. Percent change in body weight on days 1 (n = 26–30 mice per group), 2 (n = 18–21 mice per group), 3 (n = 14–16 mice per group), and 5 (n = 9 mice per group) after intranasal inoculation with 10^6^ PFU of attenuated mengovirus, vMC_0_, an equivalent amount of UV-inactivated vMC_0_, or vehicle. Data are presented as box plots. * *P*<0.001 (vMC_0_ vs. vehicle); ** *P*<0.0001, *** *P*<0.01 (vMC_0_ vs. vehicle or UV-inactivated vMC_0_); † *P*<0.01 (vMC_0_ vs. UV-inactivated vMC_0_).

Body weight reduction is a sensitive measure of viral respiratory illness in rodents [43]. Intranasal inoculation of vMC_0_ led to a significant loss of body weight on days 2 and 3 postinoculation compared with inoculation with either vehicle or UV-inactivated vMC_0_, which showed that the mice were experiencing a viral illness (Fig. 4). Mice inoculated with vMC_0_ also exhibited significant body weight loss compared with those inoculated with vehicle, but not UV-inactivated vMC_0_, on day 1 postinoculation and those inoculated with UV-inactivated vMC_0_, but not vehicle, on day 5 postinoculation (Fig. 4). Inoculation with UV-inactivated vMC_0_ resulted in no significant body weight loss compared with inoculation with vehicle, indicating a requirement for replication-competent virus for the development of illness (Fig. 4).

### Inflammation in the lower airways after inhalation of vMC_0_

**Figure 5:**
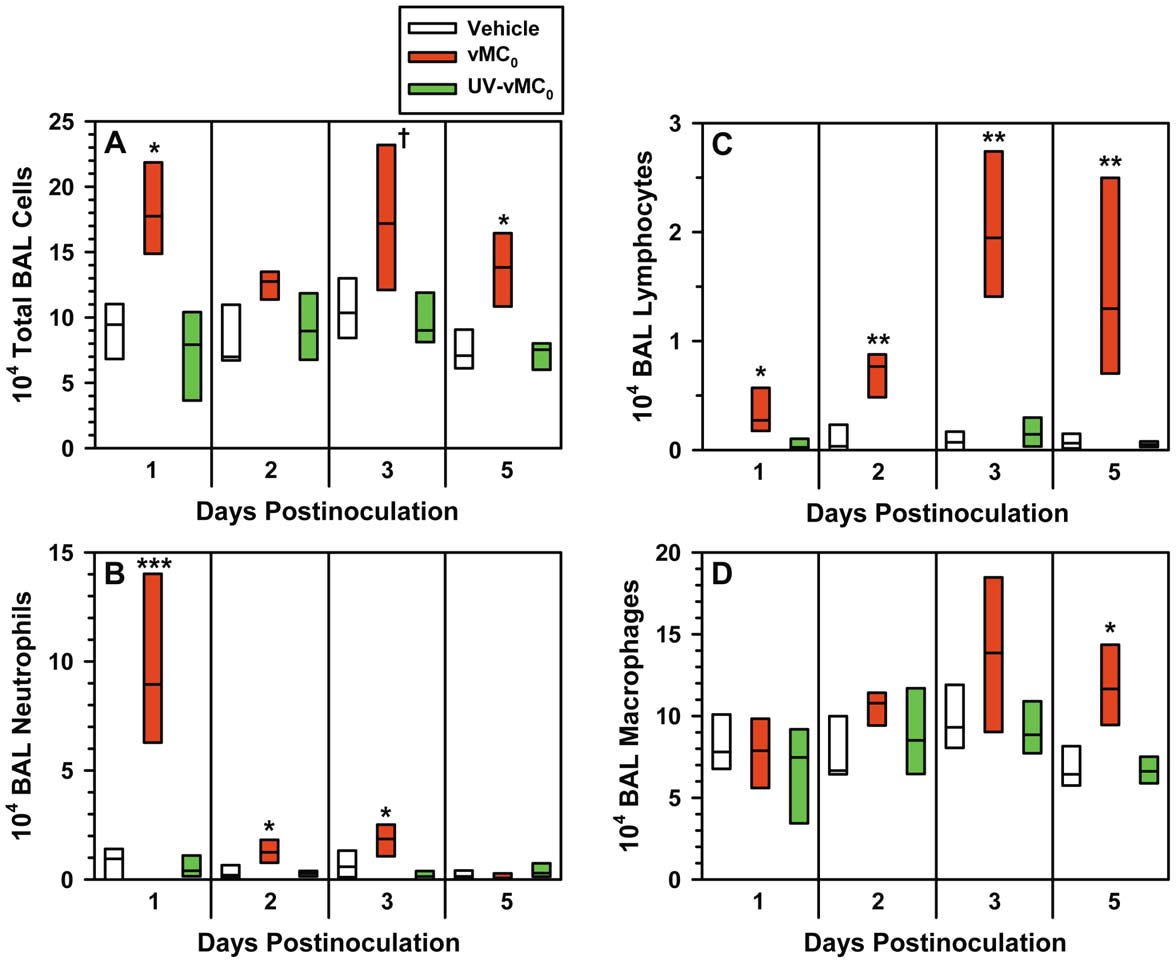
Recruitment of inflammatory cells to the lungs in response to inhalation of attenuated mengovirus. Numbers of (A) total cells, (B) neutrophils, (C) lymphocytes, and (D) macrophages in the BAL fluid harvested at the indicated times from the lungs of mice inoculated with 10^6^ PFU of vMC_0_, an equivalent amount of UV-inactivated vMC_0_, or vehicle (n = 6 mice per group). Data are presented as box plots. † *P*<0.05, * *P*<0.01, ** *P*<0.001, *** *P*<0.0001 (vMC_0_ vs. vehicle or UV-inactivated vMC_0_).

**Figure 6:**
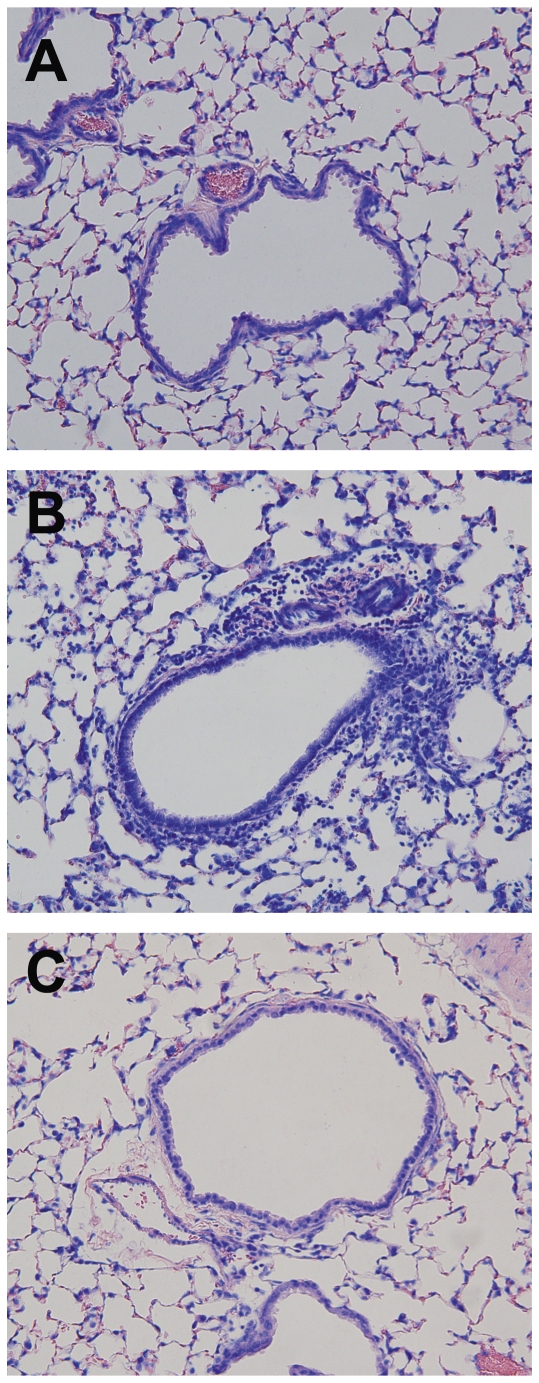
Recruitment of inflammatory cell infiltrates to the lungs in response to inhalation of attenuated mengovirus. Giemsa-stained sections of the lungs from mice intransasally inoculated with (A) vehicle, (B) vMC_0_ (10^6^ PFU) or (C) an equivalent amount of UV-inactivated vMC_0_. Lungs were harvested on day 2 postinoculation. Magnification, 20X.

Intranasal inoculation of vMC_0_ induced the recruitment of inflammatory cells into the lower airways. Total numbers of BAL cells were significantly elevated on days 1, 3, and 5 postinoculation in mice inoculated with vMC_0_ compared with those inoculated with UV-inactivated vMC_0_ or vehicle (Fig. 5A). Neutrophils (Fig. 5B) and lymphocytes (Fig. 5C) were the most prominent cell types recruited to the lower airways; numbers of BAL neutrophils and lymphocytes were significantly elevated on days 1, 2, and 3 and days 1, 2, 3, and 5 postinoculation, respectively, in vMC_0_-inoculated mice as compared with mice inoculated with vehicle or UV-inactivated vMC_0_. BAL macrophages were only significantly elevated in vMC_0_-inoculated mice on day 5 postinoculation (Fig. 5D). Few BAL eosinophils were observed, and there were no significant differences in numbers of eosinophils among the groups (not shown). Giemsa-stained lung sections revealed patchy peribronchial, perivascular, and alveolar cellular infiltrates in the lungs of mice inoculated vMC_0_ but not in those inoculated with vehicle or UV-inactivated vMC_0_ (Fig. 6).

**Figure 7:**
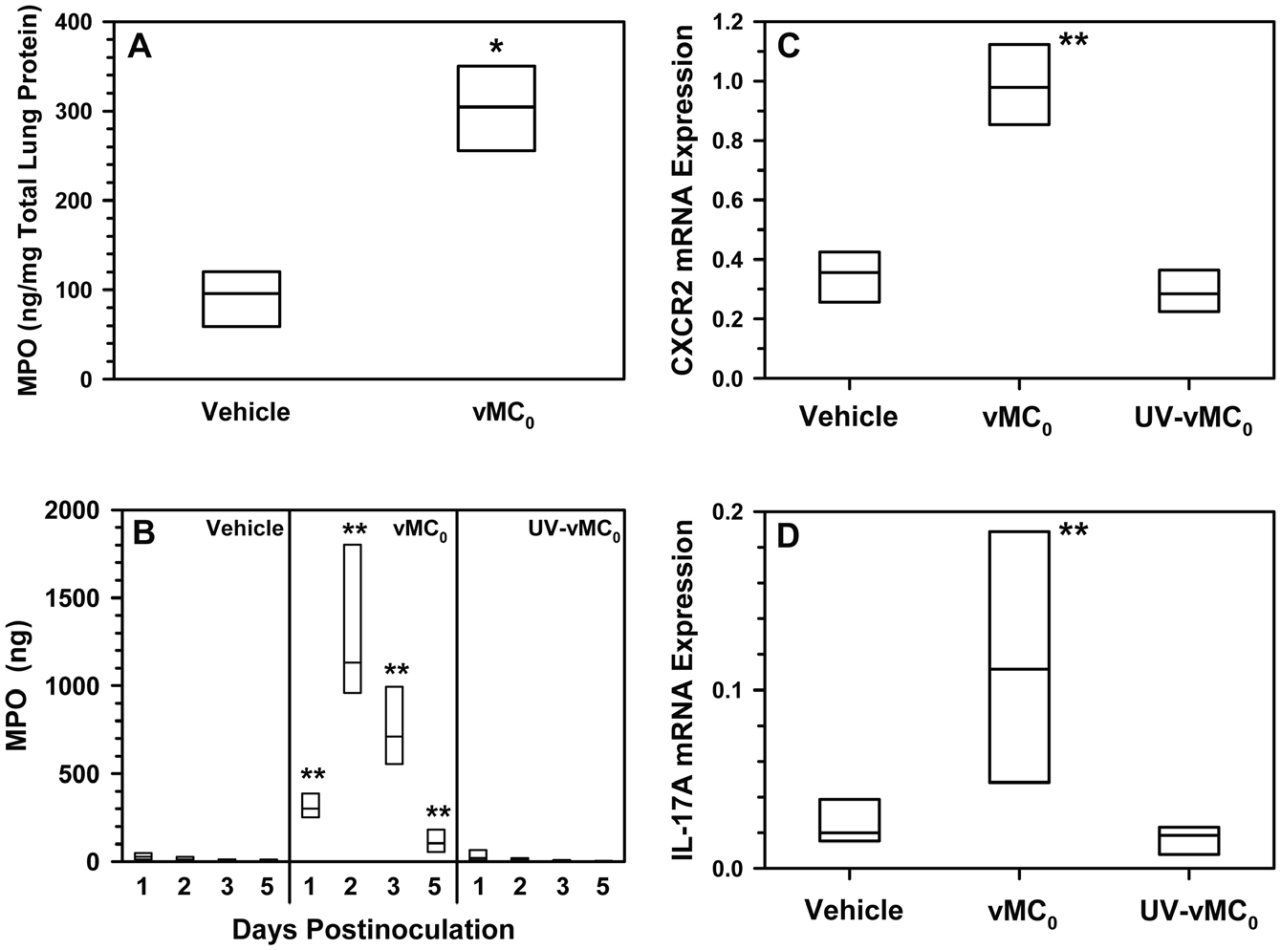
Neutrophilic inflammation in the lungs in response to inhalation of attenuated mengovirus. Mice received intranasal inoculations of 10^6^ PFU of attenuated mengovirus, vMC_0_, an equivalent amount of UV-inactivated vMC_0_, or vehicle. (A) Lung-associated MPO levels on day 1 postinoculation. MPO levels in lung tissue homogenates from mice inoculated with vehicle or vMC_0_ were determined by ELISA and normalized to total protein levels (n = 4 mice per group). (B) MPO release into airway fluids. BAL fluid was harvested on the indicated days, and MPO levels were determined by ELISA (n = 6 mice per group). Data are the total amount of MPO recovered (ELISA values were multiplied by the BAL fluid volume). MPO levels below the limit of detection were assigned a value of 1 ng for graphing purposes. (C) CXCR2 and (D) IL-17A expression in the lungs on day 1 postinoculation. Levels of CXCR2 and IL-17A mRNA were determined by real-time quantitative RT-PCR and normalized to β-actin mRNA levels (n = 6 mice per group). Data are presented as box plots. * *P*<0.05 (vMC_0_ vs. vehicle). ** *P*<0.01 (vMC_0_ vs. vehicle or UV-inactivated vMC_0_).

The levels of lung-associated neutrophil-specific granule protein, myeloperoxidase (MPO), were significantly elevated on day 1 postinoculation in vMC_0_-inoculated, compared with vehicle-inoculated, mice, indicating increased neutrophil recruitment to the lung as a whole rather than just enhanced sequestration of neutrophils into the airspace (Fig. 7A). MPO levels in the BAL fluid, a marker of neutrophil activation, were also significantly elevated on days 1, 2, 3, and 5 postinoculation in mice inoculated with vMC_0_ compared with those inoculated with UV-inactivated vMC_0_ or vehicle (Fig. 7B). CXCR2 and IL-17A mRNA levels were significantly increased on day 1 postinoculation in the lungs of mice inoculated with vMC_0_ compared with those inoculated with vehicle or UV-inactivated vMC_0_ (Fig. 7C and D). CXCR2 is the receptor on neutrophils for the neutrophil chemoattractant chemokines, known as CXCR2 ligands, and therefore serves as a useful marker for the presence of neutrophils [44]. IL-17A is an important regulator of neutrophilic inflammation [45], [46]. Along with the vMC_0_-induced increase in BAL neutrophil levels, these data demonstrate the development of neutrophilic airway inflammatory responses in the lower airways of mice after inhalation of infectious vMC_0_.

### Expression of CXCR2 ligands in the lower airways after inhalation of vMC_0_

**Figure 8:**
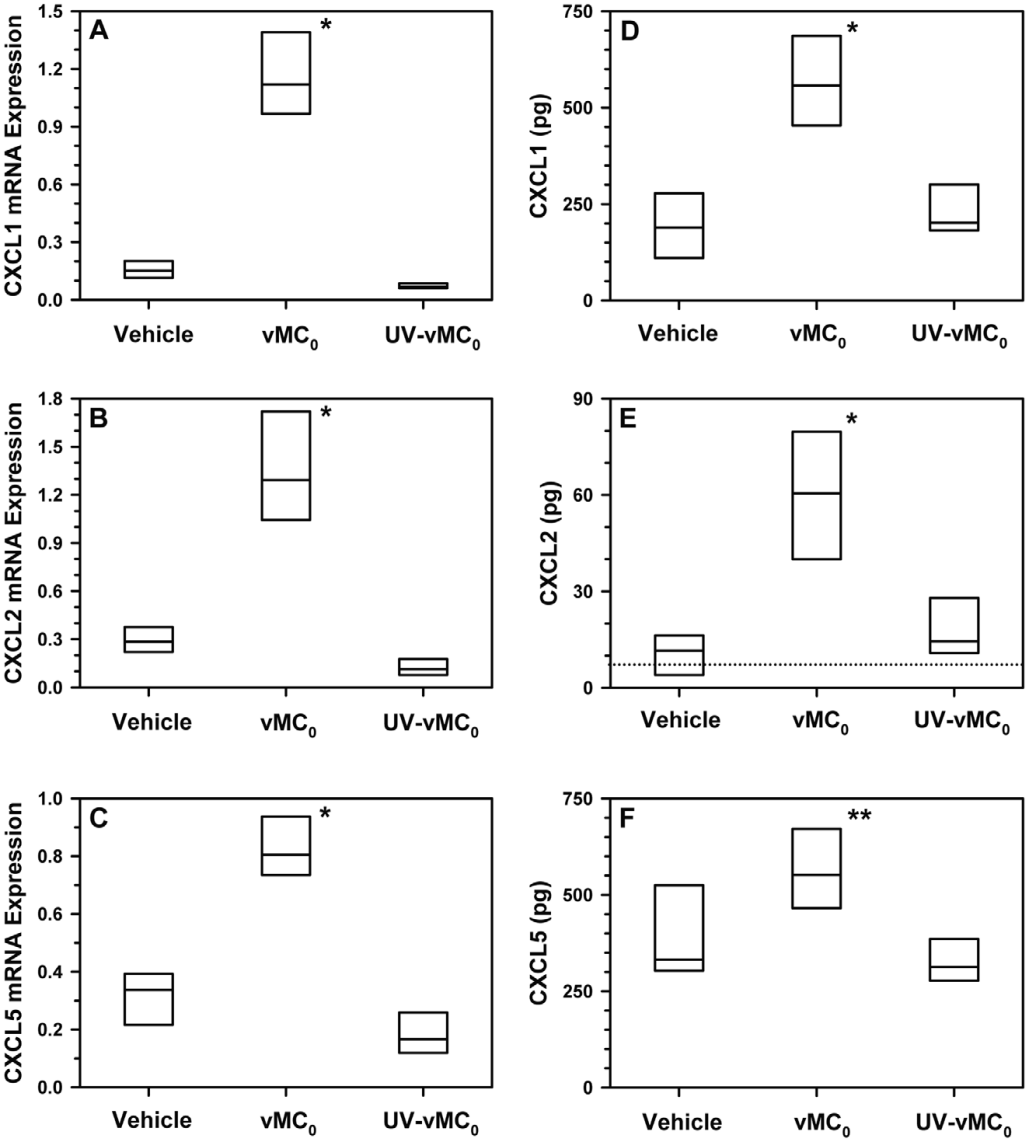
Increased pulmonary expression of CXCR2 ligands in response to inhalation of attenuated mengovirus. Mice received intranasal inoculations of 10^6^ PFU of attenuated mengovirus, vMC_0_, an equivalent amount of UV-inactivated vMC_0_, or vehicle (n = 6 mice per group). Levels of (A) CXCL1 (KC), (B) CXCL2 (MIP-2), and (C) CXCL5 (LIX) mRNA in lungs on day 1 postinoculation were determined by real-time quantitative RT-PCR and normalized to β-actin mRNA levels. Levels of (D) CXCL1, (E) CXCL2, and (F) CXCL5 protein in BAL fluid on day 2 postinoculation were determined by ELISA. Data are the total amount of chemokine recovered (ELISA values were multiplied by the BAL fluid volume). CXCL2 protein levels below the limit of detection (dotted line) were assigned a value of 4 pg for graphing purposes. Data are presented as box plots. * *P*≤0.01, ** *P*<0.05 (vMC_0_ vs. vehicle or UV-inactivated vMC_0_).

Given the significant neutrophilia induced in the lower airways by inhalation of vMC_0_, the expression of the mouse neutrophil chemoattractant CXCR2 ligands, CXCL1, CXCL2, and CXCL5, was measured. Levels of mRNA in the lung on day 1 postinoculation (Fig. 8A–C) and BAL fluid protein levels on day 2 postinoculation (Fig. 8D–F) for CXCL1, CXCL2, and CXCL5 were significantly elevated in mice inoculated with vMC_0_ in comparison with those inoculated with vehicle or UV-inactivated vMC_0_, demonstrating the availability of these chemokines to mediate neutrophil recruitment.

### Expression of CXCL10 and CCL2 in the lower airways after inhalation of vMC_0_

**Figure 9:**
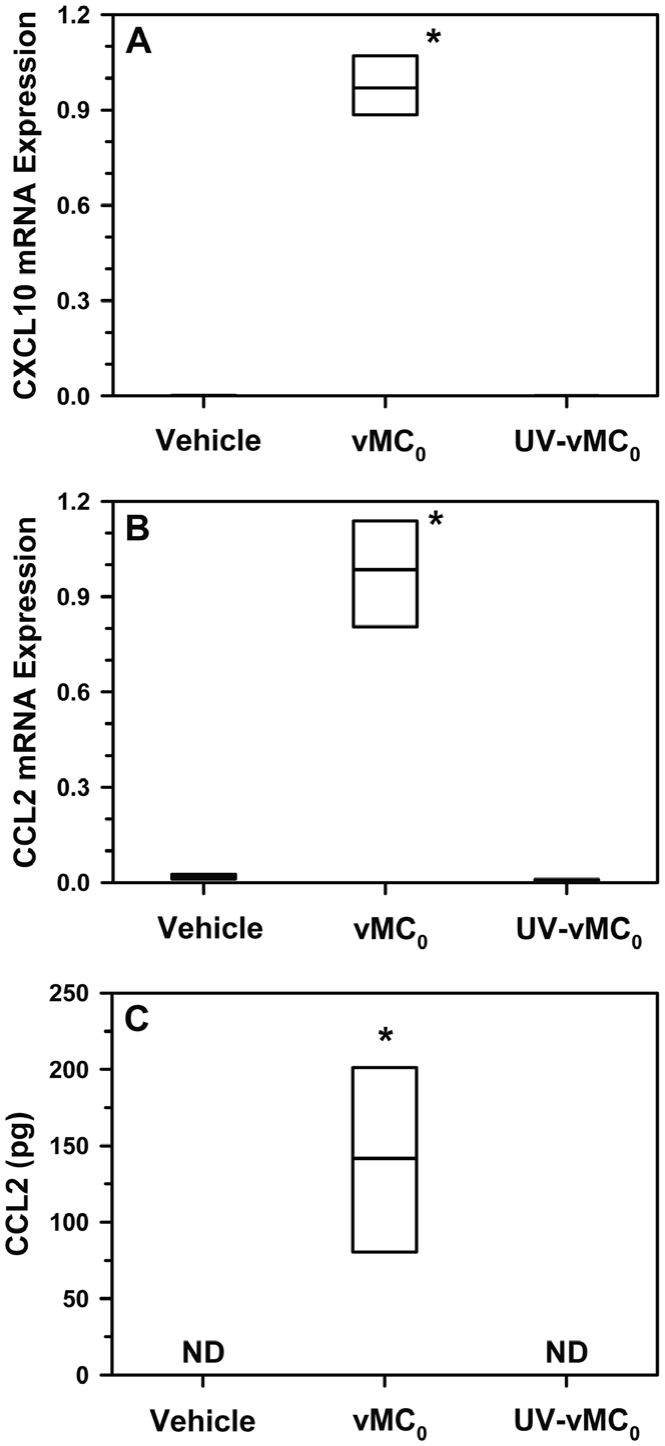
Increased pulmonary expression of CXCL10 and CCL2 in response to inhalation of attenuated mengovirus. Mice received intranasal inoculations of 10^6^ PFU of attenuated mengovirus, vMC_0_, an equivalent amount of UV-inactivated vMC_0_, or vehicle (n = 6 mice per group). Levels of (A) CXCL10 (IP-10) and (B) CCL2 (MCP-1) mRNA in lungs on day 1 postinoculation were determined by real-time quantitative RT-PCR and normalized to β-actin mRNA levels. (C) CCL2 protein levels in BAL fluid on day 2 postinoculation were determined by ELISA. Data are the total amount of CCL2 recovered (ELISA values were multiplied by the BAL fluid volume). ND, not detectable. Data are presented as box plots. * *P*<0.01 (vMC_0_ vs. vehicle or UV-inactivated vMC_0_).

Because HRV infection induces high levels of CXCL10 and CCL2 expression [47]–[50], and CCL2 indirectly contributes to neutrophil recruitment to the lungs [51], the expression of CXCL10 and CCL2 was measured. Levels of CXCL10 and CCL2 mRNA in the lungs on day 1 postinoculation and BAL fluid levels of CCL2 on day 2 postinoculation were significantly increased in vMC_0_-inoculated mice compared with vehicle- or UV-inactivated vMC_0_-inoculated mice, indicating that the picornavirus-induced expression of these chemokines in the lower airways could be studied in this mouse model (Fig. 9).

### Mucin expression in the lungs after inhalation of vMC_0_

**Figure 10:**
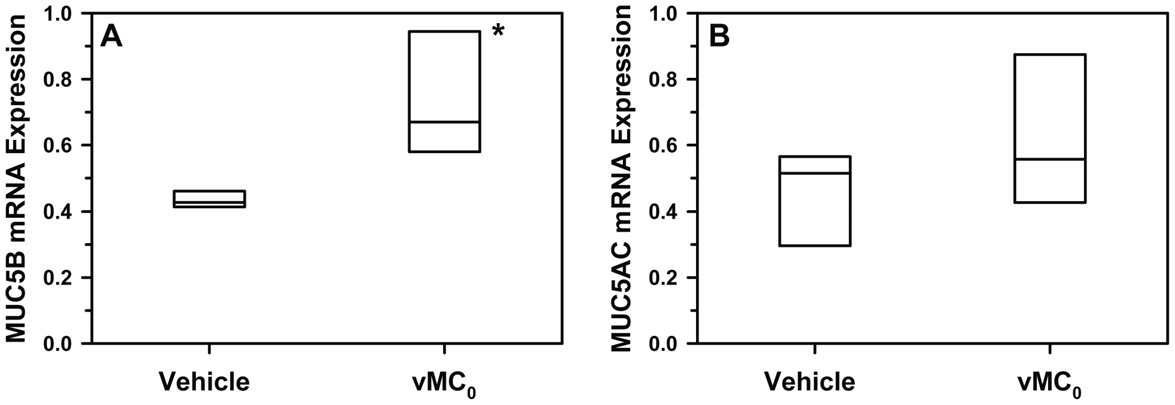
Mucin expression in the lungs in response to inhalation of attenuated mengovirus. Mice received intranasal inoculations of 10^6^ PFU of attenuated mengovirus, vMC_0_, or vehicle (n = 5 mice per group). Levels of (A) MUC5B and (B) MUC5AC mRNA in lungs on day 1 postinoculation were determined by real-time quantitative RT-PCR and normalized to β-actin mRNA levels. * *P*<0.01 (vMC_0_ vs. vehicle).

HRV infection is associated with increased mucin expression [52], [53]. Therefore, mucin mRNA expression was measured. MUC5B, but not MUC5AC, mRNA levels were significantly elevated on day 1 postinoculation in the lungs of mice inoculated with vMC_0_ as compared with vehicle-inoculated mice (Figure 10). However, periodic acid-Schiff staining of lung sections from vMC_0_-infected mice showed only sporadic staining for mucus-producing cells in the airways (not shown).

### Lung edema after inhalation of vMC_0_

**Figure 11:**
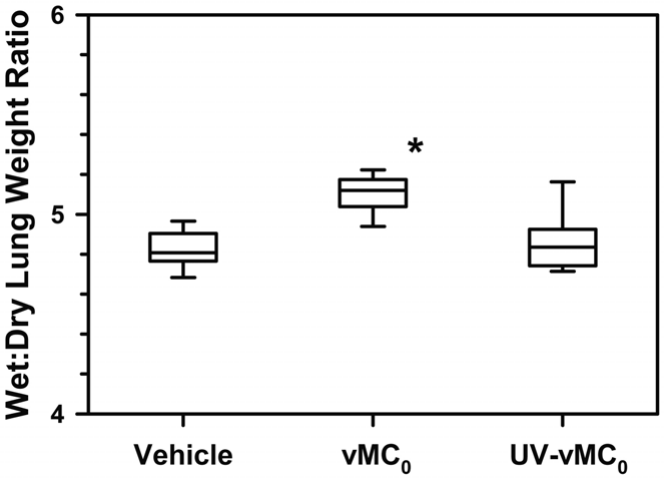
Lung edema in response to inhalation of attenuated mengovirus. Wet:dry lung weight ratios were measured for lungs harvested on day 2 after intranasal inoculation with 10^6^ PFU of attenuated mengovirus, vMC_0_, an equivalent amount of UV-inactivated vMC_0_, or vehicle (n = 9 mice per group). Data are presented as box plots; whiskers indicate the 10th and 90th percentiles. * *P*<0.01 (vMC_0_ vs. vehicle or UV-inactivated vMC_0_).

To examine whether the lower airway inflammation induced by vMC_0_ was associated with lung edema, wet:dry lung weight ratios were measured. Wet:dry lung weight ratios were significantly elevated on day 2 postinoculation in the lungs of mice inoculated with vMC_0_ as compared with vehicle- and UV-vMC_0_-inoculated mice, indicating the presence of acute lung injury (Fig. 11).

### Effect of inhalation of vMC_0_ on pulmonary physiology

**Figure 12:**
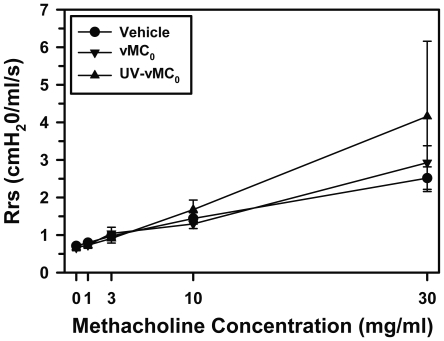
Effect of inhalation of attenuated mengovirus on pulmonary physiology. Mice received intranasal inoculations of vehicle, 10^6^ PFU of attenuated mengovirus, vMC_0_ (n = 6–7 mice per group), or an equivalent amount of UV-inactivated vMC_0_ (n = 4 mice), and on day 2 postinoculation, pulmonary physiology measurements were obtained after exposure to aerosols of normal saline followed by escalating concentrations of methacholine. Values for respiratory system resistance (Rrs) are presented as the group means ± the standard error. There were no significant differences among the groups.

To examine whether infection of the lower airways with vMC_0_ induced changes in pulmonary physiology, mice received intranasal inoculations of vMC_0_, UV-inactivated vMC_0_, or vehicle, and pulmonary function was measured on day 2 postinoculation. No significant differences were observed among the groups with regard to respiratory system resistance (Rrs) or the input impedance variables, Newtonian resistance (Rn), tissue viscance (G), and elastance (H), either at baseline or in response to methacholine challenge (Fig. 12 and data not shown), indicating a lack of viral effects on pulmonary physiology and AHR.

## Discussion

The establishment of useful and varied small animal models to study HRV pathogenesis continues to be an important goal. Diverse animal models can facilitate different types of mechanistic studies and the development of potential new therapeutic strategies [54]. Recently, murine experimental models of HRV infection have been established [23]–[28]. The development of these models represents a significant advance in the study of HRV-induced airway inflammation. However, these models have an important limitation. Although there is evidence of HRV replication in mice, HRV titers decline quickly [23]–[26]. Therefore, the development of small animal models with more robust viral replication could permit mechanistic studies of the role viral replication plays in HRV pathogenesis. To model the role of viral replication in HRV-induced lower respiratory tract infections of relevance to the development and exacerbation of asthma in humans, it would be desirable to develop small animal models where picornaviral replication occurs in the lower airways and viral titers increase, peak, and then wane while persisting for several days.

HRV is a picornavirus family member and viral replication is often most efficient in the natural hosts of a virus. Therefore, we have focused on developing small animal models using a picornavirus, an attenuated form of mengovirus, whose natural hosts are rodents. Previously, we described a rat model in which the attenuated mengovirus, vMC_0_, caused a respiratory infection in rats with several days of viral shedding accompanied by a neutrophilic lower airway inflammatory response [36]. In the mouse model described here, we observed an approximately 3 log increase in vMC_0_ titers in the lungs from 0.1 h to 24 h postinoculation, with viral shedding persisting for at least 5 days after inoculation. The intranasal inoculum of 10^6^ PFU of vMC_0_ in the mice was similar to the dose of 5×10^6^ TCID_50_ administered intranasally in HRV models in mice [23], [24]. For comparison, we inoculated mice with HRV-A01a, a minor group HRV, and found that infectious viral titers in the lungs declined until they were undetectable at 24 h postinoculation. Our HRV-A01 strain a infection data were similar to previous reports in the literature with a closely related minor receptor group virus, HRV-A01 strain b, which showed a steep reduction in HRV titers over time in the lungs of healthy, immunocompetent mice [23]–[26]. In one study, after intranasal inoculation of 5×10^6^ TCID_50_ of HRV-A01b, viral titers of approximately 10^3^, 10^3^, and 10^2^ TCID_50_/ml at 8, 16, and 24 h postinoculation, respectively, were reported [23]. In another study [25], after intranasal inoculation with 5×10^7^ TCID_50_, a 10-fold higher inoculum than used previously, lung viral titers were approximately 10^2^ and 10^1^ TCID_50_/ml on days 1 and 4 postinoculation, although induction of substantial lung injury by repeated treatment with lipopolysaccharide and elastase before virus inoculation enhanced viral titers [25]. In a third study, lung viral titers were somewhat higher after intranasal inoculation with 4.5×10^6^ TCID_50_ of HRV-A01b, with about 10^5^ PFU/ml at 4 h postinoculation steadily declining to about 10^1^ PFU/ml on day 3 postinoculation before becoming undetectable at day 4 postinoculation [26]. Overall, inhalation of HRV by mice led to some degree of viral replication accompanied by a consistent pattern of steadily declining levels of infectious virus in the lungs. In healthy, immunocompetent mice, low levels of infectious HRV shedding could be observed at 24 h postinoculation, and these levels continued to fall until little or no infectious virus was detected by day 4 postinoculation.

For both of our vMC_0_ and HRV-A01a inoculations, the amount of infectious virus detected at 0.1 h postinoculation by plaque assay was substantially lower than the inoculum dose. This discrepancy was probably due to deposition of some of the virus in the nasopharynx and gastrointestinal tract as well as the eclipsing of the virus in the lung during the early stage of the infection. Importantly, the starting levels of virus in the lungs were similar for vMC_0_ and HRV-A01a, but the viral replication and persistence were greater with vMC_0_.

.Along with the evidence of notable levels of viral replication and persistent viral shedding in the lower respiratory tract, this mouse model has many features in common with HRV infection in humans. Inhalation of live, but not inactivated vMC_0_, induced signs of illness such as significant body weight loss, which is a hallmark of viral respiratory illnesses in rodents. Infection of the lower respiratory tract with vMC_0_ resulted in the stimulation of host antiviral pathways much in the same way as HRV. There was a marked increase in Type I and III IFN proteins in the airway fluids as early as 1 day after virus inoculation, which also provides further evidence of viral replication in the lungs after vMC_0_ infection. Regulation of type I and III IFN production has been implicated as an important checkpoint in the host response to HRV infection [37]–[39]. In addition, pulmonary expression of the viral RNA sensors, TLR3, TLR7, and NOD2 were upregulated by vMC_0_ infection. The viral RNA sensors, TLR3 and TLR7, have been implicated in the host response to HRV infections [26], [40], [41]. NOD2 is an intriguing pattern recognition receptor because it not only detects bacterial peptidoglycan but can also function as a viral RNA sensor [42]. NOD2 has not been previously implicated in host responses to HRV. However, given its upregulation in the lungs in response to vMC_0_ infection, it will be of interest to examine its role in HRV infection. Depletion of neutrophils before the virus inoculation did not inhibit the subsequent upregulation of these viral RNA sensors, which was consistent with a vMC_0_-associated increase in expression in resident lung cells. In all cases, stimulation of these host antiviral pathways required active vMC_0_.

Recruitment of neutrophils and lymphocytes to the airways are common features of vMC_0_- and HRV-induced airway infections, and the chemokine milieu that was observed in the lung tissue and airway fluids in the vMC_0_-infected mice has marked similarities to that observed in HRV infections in humans. Increased pulmonary expression of the neutrophil chemoattractant CXCR2 ligands in response to vMC_0_ infection is consistent with the increased expression of CXCR2 ligands that is observed in response to HRV infection in humans and in mouse models [23], [24], [55]–[58]. We also observed the induction of pulmonary expression of CXCL10 and CCL2 in response to inhalation of vMC_0_, showing another similarity between this mouse model of picornaviral airway infection and HRV infection. CXCL10 and CCL2 expression have been shown to be markedly upregulated in response to HRV infection [23], [24], [47]–[50], [59].

There was also evidence of acute airway injury, as shown by the development of lung edema, which might be related to the neutrophilic airway inflammation observed in the lower respiratory tract and the evidence for neutrophil activation, i.e., the release of the neutrophil granule protein MPO in the airway fluids. In addition the vMC_0_ infection stimulated increased pulmonary expression of MUC5B mRNA. It is not clear why MUC5AC mRNA expression was not increased as well. In humans, HRV infection has been shown to stimulate mucin expression [52], [53]. Although we only observed sporadic staining for mucus-producing cells in the airways, it is possible that mucin gene expression induced by vMC_0_ infection might have greater biological relevance in the exacerbation of existing airway disease in mice.

Similar to what we reported in our rat model of vMC_0_-induced airway infection and inflammation [36], inhalation of vMC_0_ by mice had no significant effect on baseline pulmonary function or AHR to methacholine challenge. As in our previous rat study, experimentally naïve adult animals without existing airway disease were used in these mouse studies. Similar to our rodent models, almost all reports of experimental HRV inoculations of healthy, nonasthmatic, nonallergic human subjects have resulted in no significant postinoculation changes in baseline pulmonary function or AHR [11], [60]–[64]. One study showed small differences in AHR after experimental HRV infection of nonasthmatic, nonallergic subjects, but these small changes were only detectable at a methacholine concentration that was a half-log above the upper end of the typical dose range [65]. However, experimental HRV inoculation was shown to increase AHR in some individuals with asthma and/or allergic rhinitis in several studies [11], [22], [64], [66], [67] but not in others [60], [61], [63], [65], [68]. Consequently, the lack of AHR changes in healthy adult mice without existing airway disease was consistent with outcomes of experimental HRV infections in humans with no underlying airway disease, such as asthma or allergic rhinitis. Overall, the absence of pulmonary function changes during vMC_0_-induced lower respiratory infection in mice without existing airway disease is consistent with observations in experimental HRV infections in healthy human volunteers. The absence of viral effects on AHR is also consistent with one mouse model of HRV infection in which no increase in AHR to methacholine challenge was observed after HRV infection unless the BALB/c mice had underlying experimental allergic airway inflammation [23]. However, in another model, a modest increase in AHR to methacholine challenge was reported after infection of C57BL/6 mice with HRV [24], which might be related to mouse strain differences. The underlying condition of the airways, whether related to genetic, developmental, and/or environmental factors, may have profound effects on host responses to picornaviral airway infection and the outcomes of these infections. In the future, it will be of interest to investigate the influence of age, genetic factors, and other environmental exposures, such as allergens, other respiratory viruses [69], or microbial factors, on host responses to vMC_0_-induced lower respiratory tract infection.

A potential limitation of using vMC_0_ in this mouse model of respiratory infection is that, unlike HRV, mengovirus is neurotropic. However, it is important to note that the HRV, as enteroviruses, are closely related to poliovirus, which is also neurotropic. Inhalation of the attenuated mutant of mengovirus, vMC_0_, induced a self-limited respiratory infection in rodents, demonstrating the plasticity of vMC_0_ with regard to its tissue tropism. After intranasal inoculation with vMC_0_, there was no evidence of systemic infection in homogenates of nonrespiratory organs, as tested by plaque assay (not shown), which was consistent with our previous work in rats [36]. We chose to use plaque assays for the detection of virus because our primary interest was in measuring titers of infectious virus. We cannot rule out the possibility that vMC_0_ RNA might have been detected in nonrespiratory organs if a highly sensitive PCR-based assay had been used. In fact, HRV viremia has been detected in some human patients by highly sensitive PCR-based assays [70], [71].

The picornaviral airway infection models in rodents involve a single viral inoculation, which is comparable to experimental HRV inoculation studies in human subjects. An HRV infection can trigger a wheezing illness or asthma exacerbation in children and adults [1], and experiencing an HRV wheezing illness early in life is associated with an increased risk for the subsequent development of childhood asthma [2]–[4]. Thus, these rodent models should be useful for relevant mechanistic studies. Some children appear to exhibit increased susceptibility to recurrent HRV infections, presumably caused by exposure to differing serotypes [72], [73], but the precise role of recurrent HRV infections in asthma pathogenesis remains to be elucidated. Recurrent picornaviral airway infections would be more challenging to study in animal models because of the limited serotypic diversity of viruses that have been experimentally tested in mice, but one possibility would involve sequential inoculation of vMC_0_ and HRV because there should be limited immune crossreactivity between these picornaviruses.

A comparison between our mengovirus infection model and the HRV infection model in mice [23]–[28] shows that they both induce a neutrophilic inflammatory response in the lower respiratory tract, which is consistent with the inflammatory responses induced by HRV infections in humans. By mimicking important aspects of natural HRV infections in humans, both models should facilitate mechanistic studies that would be difficult to perform in human subjects. The HRV infection model in mice allows for the effects of the actual human virus to be studied directly, but HRV is not a natural mouse pathogen and does not replicate efficiently in mice. An important distinction is that mengovirus, a natural mouse pathogen, replicates more efficiently in mice than does HRV. Our data show that after inhalation of vMC_0_, viral titers in the lung increase, peak, and then wane while persisting for at least 5 days. Overall, both of these models have strengths and limitations, and the relative usefulness of each model will depend on the specific research questions that are being addressed.

In conclusion, we have developed of a robust model of picornavirus-induced airway infection and inflammation in mice. One of this model's key strengths is that it employs a natural pathogen for rodents, mengovirus, which yields viral replication kinetics and magnitudes closer to natural viral respiratory infections. HRV infection models in mice are proving to be useful for the study of HRV-induced airway inflammation but have a weaker component of viral replication. The mengovirus airway infection model should complement these existing models, and should be especially useful for studies where viral replication is an important outcome.

## Materials and Methods

### Ethics statement

The mice were housed and all experimental procedures were performed in an American Association for Accreditation of Laboratory Animal Care-accredited laboratory animal facility at the University of Wisconsin School of Medicine and Public Health. The study was approved by the University of Wisconsin School of Medicine and Public Health Animal Care and Use Committee (protocol number M00582) and conformed to the Guide for the Care and Use of Laboratory Animals.

### Animals

Female BALB/cAnNCr mice were purchased from the National Cancer Institute Animal Production Program (Frederick, MD) and used at 6–10 weeks of age for inoculation studies. The mice were housed in microisolator cages within HEPA-filtered isolation cubicles (Britz & Company, Wheatland, WY).

### Virus

Recombinant vMC_0_ has been described previously [31]. To prepare stocks of vMC_0_, the plasmid containing the entire vMC_0_ cDNA was linearized and transcribed *in vitro* with T7 RNA polymerase. The viral RNA was transfected into HeLa cells, and plaques were isolated and eluted overnight. The viral plaque eluate was used to inoculate fresh plates of HeLa cells which were incubated for 24 h at 37°C. The plates were then frozen, thawed, and scraped. The lysate underwent two additional freeze-thaw cycles before centrifugation to remove cellular debris. This lysate was used to inoculate HeLa cells (multiplicity of infection = 1) grown in suspension to a density of 4×10^8^ cells/ml. Flasks were incubated at 37°C for 9 h in a shaking water bath and then frozen at −80°C. Viral stocks were concentrated and purified by centrifugation through a 30% sucrose cushion. Titers were determined by duplicate plating on HeLa cell monolayers [74]. UV-inactivated vMC_0_ stocks were prepared by exposing the virus to a germicidal UV lamp for 30 min. Plaque assays using HeLa cells were employed to verify that active virus was undetectable [<10 PFU/ml] in the UV-inactivated preparations. Purified HRV-A, genotype 1 (A01a) was provided by Dr. Wai-Ming Lee (University of Wisconsin-Madison, Madison, WI).

### Virus inoculation and measurement of viral titers

Mice were inoculated intranasally with vehicle, 10^6^ PFU of vMC_0_, 10^6^ PFU equivalents of UV-inactivated vMC_0_, or 5×10^6^ PFU of HRV-A01a under isoflurane anesthesia. For virus titration, lungs were removed from the chest cavity aseptically and weighed. The lungs were homogenized with an automated tissue homogenizer in an appropriate volume of phosphate-buffered saline (PBS) to yield a 10% w/v homogenate. Cell debris were removed by centrifugation, and the supernates were titered for virus by duplicate plating on HeLa cell monolayers [74].

### Depletion of neutrophils

Purified anti-neutrophil mAb 1A8 (mouse Gr-1/Ly-6G-specific) and isotype-matched control mAb 2A3 (rat IgG2a; <0.18 and <0.63 endotoxin units/mg by Limulus amebocyte lysate test, respectively) were purchased from Bio X Cell (West Lebanon, NH). Anti-neutrophil mAb was administered to each mouse by both intraperitoneal and intranasal routes, which has been previously shown to effectively deplete neutrophils in the respiratory tract [75]. However, we used mAb 1A8 to deplete neutrophils rather than mAb RB6-8C5 because of its greater selectivity for neutrophils [76]. 1A8 was effective in depleting neutrophils, as a single intraperitoneal injection of 1A8 (500 µg) the day before virus inoculation reduced the day 1 postinoculation vMC_0_-induced increase in lung MPO protein levels by 81% in comparison with mice receiving 2A3 (500 µg) (n = 4 mice per group). One day before intranasal inoculation with either vehicle or 10^6^ PFU of vMC_0_, mice received intraperitoneal injections of 500 µg and intranasal instillations (under isoflurane anesthesia) of 200 µg of either 1A8 or 2A3 in PBS. On the day of inoculation, the mice received an additional intraperitoneal injection of 500 µg of either 1A8 or 2A3 in PBS.

### Measurements of pulmonary inflammation

Mice were anesthetized and exsanguinated by severing the dorsal aorta. After opening the chest, BAL was performed. The lungs were lavaged with 800 µl of PBS via a tracheal catheter. The lungs were then lavaged again in the same manner, and the BAL fluid samples were combined. The BAL fluid was centrifuged, and the cell pellet was resuspended in 0.2 ml of PBS. The total number of BAL leukocytes was determined with an automated cell counter (model Z1, Beckman Coulter, Hialeah, FL). Differential cell counts were determined by counting 200 leukocytes on cytospin slides stained with Protocol® Wright-Giemsa stain according to the manufacturer's protocol (Fisher Diagnostics, Middletown, VA). BAL fluid was stored at −80°C. For histological assessments of pulmonary inflammation, mouse lungs were filled with 10% formalin, tied off, removed from the chest cavity, and immersed in 10% formalin for 24 h. The tissue was then processed, embedded in paraffin, and cut into 5 µm sections. Sections were stained with a Giemsa stain.

### Measurements of pulmonary protein expression

The levels of specific proteins in the BAL fluid or lung homogenates were measured by enzyme-linked immunosorbent assay (ELISA). Mouse CXCL1 (KC, keratinocyte-derived cytokine)-specific, CXCL2 (MIP-2, macrophage inflammatory protein-2)-specific, and IFN-β-specific ELISA kits with sensitivities of 7.8 pg/ml and Mouse IFN-α-specific kits with a sensitivity of 12.5 pg/ml were purchased from Invitrogen (Carlsbad, CA). Mouse CXCL5 (LIX, lipopolysaccharide-induced CXC chemokine)-specific and IFN-λ-specific ELISA kits with sensitivities of 7.8 pg/ml were acquired from R&D Systems (Minneapolis, MN). Mouse CCL2 (MCP-1, monocyte chemoattractant protein-1)-specific ELISA kits with a sensitivity of 7.8 pg/ml were purchased from BD Biosciences (San Diego, CA). Mouse MPO-specific ELISA kits with a sensitivity of 0.4 ng/ml were obtained from Cell Sciences Incorporated (Canton, MA). Total protein levels in lung homogenates were measured by the Coomassie Plus (Bradford) Protein Assay (Pierce, Rockford, IL).

### Measurements of pulmonary gene expression

For RNA isolation, mouse lungs were harvested in an RNase-free manner and immediately flash-frozen in liquid nitrogen. Lungs were then powdered under liquid nitrogen with a chilled mortar and pestle. Total RNA was isolated from frozen lung powder with an RNeasy Mini Kit according to the manufacturer's instructions (Qiagen, Valencia, CA). The total RNA was reverse transcribed into cDNA with SuperScript® III reverse transcriptase following the manufacturer's instructions (Invitrogen, Carlsbad, CA). The cDNA product was diluted 1∶2.5 fold and used as the template for quantitative real-time PCR, which was performed using an ABI 7500 Real Time PCR System (Applied Biosystems, Foster City, CA). TaqMan® primer and probe sets for TLR3, TLR7, NOD2, CXCL1, CXCL2, CXCL5, CXCL10 (IP-10, IFN-gamma inducible protein 10), CCL2, MUC5B, MUC5AC, and β-actin were purchased from Applied Biosystems. All values were normalized to the endogenous control, β-actin. Standard curves were generated by serial dilutions of cDNA from the lungs of a vMC_0_-inoculated mouse and used to determine relative expression levels.

### Measurement of lung edema

To measure lung edema, wet:dry lung weight ratios were determined. Lungs were removed from the chest cavity and immediately weighed to obtain the wet lung weight. All lungs were place in an oven at 65°C for 4 days and then weighed again to determine the dry lung weight.

### Measurements of pulmonary physiology

Mice were anesthetized with pentobarbital (Abbott, North Chicago, IL), intubated via tracheostomy, paralyzed with succinylcholine HCl (Sigma, St. Louis, MO), and ventilated mechanically (flexiVent, SCIREQ, Montreal, QC, Canada). Aerosol challenges were delivered by the ventilator via an inline nebulizer (Aeroneb, SCIREQ) with aerosolized normal saline being followed by methacholine HCl (Sigma) solutions in concentrations of 1, 3, 10, and 30 mg/ml. After each aerosol challenge, measurements of pulmonary physiology were performed by the flexiVent system, alternating measures of Rrs with measures of input impedance variables (Rn, G, and H). For each variable, the highest value occurring after each aerosol challenge was recorded as the response, referenced to the value obtained after saline challenge.

### Statistical analysis

The numbers of total cells, neutrophils, lymphocytes, and macrophages in the BAL fluid were analyzed by an analysis of variance (general linear model), which was followed by planned pairwise comparisons using Fischer's least significant difference test. A residual analysis was employed to test the adequacy of the models. Pulmonary physiology data were analyzed by an analysis of covariance, using the values from the methacholine dose 30 mg/ml as the dependent variable and baseline (after saline) values as the covariate to account for differences in baselines. Nonparametric tests were used to analyze all other data. The Kruskal-Wallis test was used for comparisons among three or more groups and was followed by planned pairwise comparisons using the Mann-Whitney test. For comparisons between two groups, the Mann-Whitney test was used. Box plots depict the median and the interquartile range between the 25th and 75th percentile, and whiskers show the 10th and 90th percentiles. Analyses were performed using the statistical software package SYSTAT 11.0 (Systat Software, Chicago, IL).

## References

[1] Busse WW, Lemanske RF, Gern JE (2010). Role of viral respiratory infections in asthma and asthma exacerbations.. Lancet.

[2] Lemanske RF, Jackson DJ, Gangnon RE, Evans MD, Li Z (2005). Rhinovirus illnesses during infancy predict subsequent childhood wheezing.. J Allergy Clin Immunol.

[3] Jackson DJ, Gangnon RE, Evans MD, Roberg KA, Anderson EL (2008). Wheezing rhinovirus illnesses in early life predict asthma development in high-risk children.. Am J Respir Crit Care Med.

[4] Kusel MM, de Klerk NH, Kebadze T, Vohma V, Holt PG (2007). Early-life respiratory viral infections, atopic sensitization, and risk of subsequent development of persistent asthma.. J Allergy Clin Immunol.

[5] Rosenthal LA, Avila PC, Heymann PW, Martin RJ, Miller EK (2010). Viral respiratory tract infections and asthma: the course ahead.. J Allergy Clin Immunol.

[6] Singh AM, Moore PE, Gern JE, Lemanske RF, Hartert TV (2007). Bronchiolitis to asthma: a review and call for studies of gene-virus interactions in asthma causation.. Am J Respir Crit Care Med.

[7] Gern JE, Galagan DM, Jarjour NN, Dick EC, Busse WW (1997). Detection of rhinovirus RNA in lower airway cells during experimentally induced infection.. Am J Respir Crit Care Med.

[8] Papadopoulos NG, Bates PJ, Bardin PG, Papi A, Leir SH (2000). Rhinoviruses infect the lower airways.. J Infect Dis.

[9] Mosser AG, Brockman-Schneider R, Amineva S, Burchell L, Sedgwick JB (2002). Similar frequency of rhinovirus-infectible cells in upper and lower airway epithelium.. J Infect Dis.

[10] Schroth MK, Grimm E, Frindt P, Galagan DM, Konno SI (1999). Rhinovirus replication causes RANTES production in primary bronchial epithelial cells.. Am J Respir Cell Mol Biol.

[11] Message SD, Laza-Stanca V, Mallia P, Parker HL, Zhu J (2008). Rhinovirus-induced lower respiratory illness is increased in asthma and related to virus load and Th1/2 cytokine and IL-10 production.. Proc Natl Acad Sci U S A.

[12] Mosser AG, Vrtis R, Burchell L, Lee WM, Dick CR (2005). Quantitative and qualitative analysis of rhinovirus infection in bronchial tissues.. Am J Respir Crit Care Med.

[13] Malmstrom K, Pitkaranta A, Carpen O, Pelkonen A, Malmberg LP (2006). Human rhinovirus in bronchial epithelium of infants with recurrent respiratory symptoms.. J Allergy Clin Immunol.

[14] Wos M, Sanak M, Soja J, Olechnowicz H, Busse WW (2008). The presence of rhinovirus in lower airways of patients with bronchial asthma.. Am J Respir Crit Care Med.

[15] Kelly JT, Busse WW (2008). Host immune responses to rhinovirus: mechanisms in asthma.. J Allergy Clin Immunol.

[16] Jarjour NN, Gern JE, Kelly EA, Swenson CA, Dick CR (2000). The effect of an experimental rhinovirus 16 infection on bronchial lavage neutrophils.. J Allergy Clin Immunol.

[17] Gern JE, Vrtis R, Grindle KA, Swenson C, Busse WW (2000). Relationship of upper and lower airway cytokines to outcome of experimental rhinovirus infection.. Am J Respir Crit Care Med.

[18] Denlinger LC, Shi L, Guadarrama A, Schell K, Green D (2009). Attenuated P2X7 pore function as a risk factor for virus-induced loss of asthma control.. Am J Respir Crit Care Med.

[19] Fahy JV, Kim KW, Liu J, Boushey HA (1995). Prominent neutrophilic inflammation in sputum from subjects with asthma exacerbation.. J Allergy Clin Immunol.

[20] Wark PA, Johnston SL, Moric I, Simpson JL, Hensley MJ (2002). Neutrophil degranulation and cell lysis is associated with clinical severity in virus-induced asthma.. Eur Respir J.

[21] Gern JE, Martin MS, Anklam KA, Shen K, Roberg KA (2002). Relationships among specific viral pathogens, virus-induced interleukin- 8, and respiratory symptoms in infancy.. Pediatr Allergy Immunol.

[22] Grunberg K, Timmers MC, Smits HH, de Klerk EP, Dick EC (1997). Effect of experimental rhinovirus 16 colds on airway hyperresponsiveness to histamine and interleukin-8 in nasal lavage in asthmatic subjects in vivo.. Clin Exp Allergy.

[23] Bartlett NW, Walton RP, Edwards MR, Aniscenko J, Caramori G (2008). Mouse models of rhinovirus-induced disease and exacerbation of allergic airway inflammation.. Nat Med.

[24] Newcomb DC, Sajjan US, Nagarkar DR, Wang Q, Nanua S (2008). Human rhinovirus 1B exposure induces phosphatidylinositol 3-kinase-dependent airway inflammation in mice.. Am J Respir Crit Care Med.

[25] Sajjan U, Ganesan S, Comstock AT, Shim J, Wang Q (2009). Elastase- and LPS-exposed mice display altered responses to rhinovirus infection.. Am J Physiol Lung Cell Mol Physiol.

[26] Wang Q, Miller DJ, Bowman ER, Nagarkar DR, Schneider D (2011). MDA5 and TLR3 Initiate Pro-Inflammatory Signaling Pathways Leading to Rhinovirus-Induced Airways Inflammation and Hyperresponsiveness.. PLoS Pathog.

[27] Nagarkar DR, Wang Q, Shim J, Zhao Y, Tsai WC (2009). CXCR2 is required for neutrophilic airway inflammation and hyperresponsiveness in a mouse model of human rhinovirus infection.. J Immunol.

[28] Nagarkar DR, Bowman ER, Schneider D, Wang Q, Shim J (2010). Rhinovirus infection of allergen-sensitized and -challenged mice induces eotaxin release from functionally polarized macrophages.. J Immunol.

[29] Palmenberg AC, Osorio JE (1994). Cardioviral poly(C) tracts and viral pathogenesis.. Arch Virol Suppl.

[30] Duke GM, Osorio JE, Palmenberg AC (1990). Attenuation of Mengo virus through genetic engineering of the 5′ noncoding poly(C) tract.. Nature.

[31] Martin LR, Duke GM, Osorio JE, Hall DJ, Palmenberg AC (1996). Mutational analysis of the mengovirus poly(C) tract and surrounding heteropolymeric sequences.. J Virol.

[32] Osorio JE, Grossberg SE, Palmenberg AC (2000). Characterization of genetically engineered mengoviruses in mice.. Viral Immunol.

[33] Osorio JE, Martin LR, Palmenberg AC (1996). The immunogenic and pathogenic potential of short poly(C) tract Mengo viruses.. Virology.

[34] Martin LR, Neal ZC, McBride MS, Palmenberg AC (2000). Mengovirus and encephalomyocarditis virus poly(C) tract lengths can affect virus growth in murine cell culture.. J Virol.

[35] Gern JE, Dick EC, Lee WM, Murray S, Meyer K (1996). Rhinovirus enters but does not replicate inside monocytes and airway macrophages.. J Immunol.

[36] Rosenthal LA, Amineva SP, Szakaly RJ, Lemanske RF, Gern JE (2009). A rat model of picornavirus-induced airway infection and inflammation.. Virol J.

[37] Wark PA, Johnston SL, Bucchieri F, Powell R, Puddicombe S (2005). Asthmatic bronchial epithelial cells have a deficient innate immune response to infection with rhinovirus.. J Exp Med.

[38] Contoli M, Caramori G, Mallia P, Johnston S, Papi A (2005). Mechanisms of respiratory virus-induced asthma exacerbations.. Clin Exp Allergy.

[39] Cakebread JA, Xu Y, Grainge C, Kehagia V, Howarth PH (2011). Exogenous IFN-beta has antiviral and anti-inflammatory properties in primary bronchial epithelial cells from asthmatic subjects exposed to rhinovirus.. J Allergy Clin Immunol.

[40] Slater L, Bartlett NW, Haas JJ, Zhu J, Message SD (2010). Co-ordinated role of TLR3, RIG-I and MDA5 in the innate response to rhinovirus in bronchial epithelium.. PLoS Pathog.

[41] Kuo C, Lim S, King NJ, Johnston SL, Burgess JK (2011). Rhinovirus infection induces extracellular matrix protein deposition in asthmatic and nonasthmatic airway smooth muscle cells.. Am J Physiol Lung Cell Mol Physiol.

[42] Sabbah A, Chang TH, Harnack R, Frohlich V, Tominaga K (2009). Activation of innate immune antiviral responses by Nod2.. Nat Immunol.

[43] Sorkness RL, Castleman WL, Kumar A, Kaplan MR, Lemanske RF (1999). Prevention of chronic post-bronchiolitis airway sequelae with interferon-γ treatment in rats.. Am J Respir Crit Care Med.

[44] Stillie R, Farooq SM, Gordon JR, Stadnyk AW (2009). The functional significance behind expressing two IL-8 receptor types on PMN.. J Leukoc Biol.

[45] Wiehler S, Proud D (2007). Interleukin-17A modulates human airway epithelial responses to human rhinovirus infection.. Am J Physiol Lung Cell Mol Physiol.

[46] Nembrini C, Marsland BJ, Kopf M (2009). IL-17-producing T cells in lung immunity and inflammation.. J Allergy Clin Immunol.

[47] Spurrell JC, Wiehler S, Zaheer RS, Sanders SP, Proud D (2005). Human airway epithelial cells produce IP-10 (CXCL10) in vitro and in vivo upon rhinovirus infection.. Am J Physiol Lung Cell Mol Physiol.

[48] Korpi-Steiner NL, Bates ME, Lee WM, Hall DJ, Bertics PJ (2006). Human rhinovirus induces robust IP-10 release by monocytic cells, which is independent of viral replication but linked to type I interferon receptor ligation and STAT1 activation.. J Leukoc Biol.

[49] Korpi-Steiner NL, Valkenaar SM, Bates ME, Evans MD, Gern JE (2010). Human monocytic cells direct the robust release of CXCL10 by bronchial epithelial cells during rhinovirus infection.. Clin Exp Allergy.

[50] Hall DJ, Bates ME, Guar L, Cronan M, Korpi N (2005). The role of p38 MAPK in rhinovirus-induced monocyte chemoattractant protein-1 production by monocytic-lineage cells.. J Immunol.

[51] Maus UA, Waelsch K, Kuziel WA, Delbeck T, Mack M (2003). Monocytes are potent facilitators of alveolar neutrophil emigration during lung inflammation: role of the CCL2-CCR2 axis.. J Immunol.

[52] Yuta A, Doyle WJ, Gaumond E, Ali M, Tamarkin L (1998). Rhinovirus infection induces mucus hypersecretion.. Am J Physiol.

[53] Hewson CA, Haas JJ, Bartlett NW, Message SD, Laza-Stanca V (2010). Rhinovirus induces MUC5AC in a human infection model and in vitro via NF-kappaB and EGFR pathways.. Eur Respir J.

[54] Rosenthal LA (2010). Animal models of virus-induced chronic airway disease.. Immunol Allergy Clin North Am.

[55] Zhu Z, Tang W, Gwaltney JM, Wu Y, Elias JA (1997). Rhinovirus stimulation of interleukin-8 in vivo and in vitro: role of NF-kappaB.. Am J Physiol.

[56] Subauste MC, Jacoby DB, Richards S, Proud D (1995). Infection of a human respiratory epithelial cell line with rhinovirus. Induction of cytokine release and modulation of susceptibility to infection by cytokine exposure.. J Clin Invest.

[57] Donninger H, Glashoff R, Haitchi HM, Syce JA, Ghildyal R (2003). Rhinovirus induction of the CXC chemokine epithelial-neutrophil activating peptide-78 in bronchial epithelium.. J Infect Dis.

[58] Bochkov YA, Hanson KM, Keles S, Brockman-Schneider RA, Jarjour NN (2010). Rhinovirus-induced modulation of gene expression in bronchial epithelial cells from subjects with asthma.. Mucosal Immunol.

[59] DeMore JP, Weisshaar EH, Vrtis RF, Swenson CA, Evans MD (2009). Similar colds in subjects with allergic asthma and nonatopic subjects after inoculation with rhinovirus-16.. J Allergy Clin Immunol.

[60] Skoner DP, Doyle WJ, Seroky J, Vandeusen MA, Fireman P (1996). Lower airway responses to rhinovirus 39 in healthy allergic and nonallergic subjects.. Eur Respir J.

[61] Zambrano JC, Carper HT, Rakes GP, Patrie J, Murphy DD (2003). Experimental rhinovirus challenges in adults with mild asthma: Response to infection in relation to IgE.. J Allergy Clin Immunol.

[62] de KJ, Grunberg K, Sont JK, Hoogeveen M, van Schadewijk WA (2002). Rhinovirus infection in nonasthmatic subjects: effects on intrapulmonary airways.. Eur Respir J.

[63] Angelini B, Van Deusen MA, Doyle WJ, Seroky J, Cohen S (1997). Lower airway responses to rhinovirus-Hanks in healthy subjects with and without allergy.. J Allergy Clin Immunol.

[64] Gern JE, Calhoun W, Swenson C, Shen G, Busse WW (1997). Rhinovirus infection preferentially increases lower airway responsiveness in allergic subjects.. Am J Respir Crit Care Med.

[65] Fleming HE, Little FF, Schnurr D, Avila PC, Wong H (1999). Rhinovirus-16 colds in healthy and in asthmatic subjects: similar changes in upper and lower airways.. Am J Respir Crit Care Med.

[66] Lemanske RF, Dick EC, Swenson CA, Vrtis RF, Busse WW (1989). Rhinovirus upper respiratory infection increases airway hyperreactivity and late asthmatic reactions.. J Clin Invest.

[67] de Gouw HW, Grunberg K, Schot R, Kroes AC, Dick EC (1998). Relationship between exhaled nitric oxide and airway hyperresponsiveness following experimental rhinovirus infection in asthmatic subjects.. Eur Respir J.

[68] Avila PC, Abisheganaden J, Wong H, Liu J, Yagi S (2000). Effects of allergic inflammation of the nasal mucosa on the severity of rhinovirus 16 cold.. J Allergy Clin Immunol.

[69] Sorkness RL, Herricks KM, Szakaly RJ, Lemanske RF, Rosenthal LA (2007). Altered allergen-induced eosinophil trafficking and physiological dysfunction in airways with preexisting virus-induced injury.. Am J Physiol Lung Cell Mol Physiol.

[70] Xatzipsalti M, Kyrana S, Tsolia M, Psarras S, Bossios A (2005). Rhinovirus viremia in children with respiratory infections.. Am J Respir Crit Care Med.

[71] Fuji N, Suzuki A, Lupisan S, Sombrero L, Galang H (2011). Detection of Human Rhinovirus C Viral Genome in Blood among Children with Severe Respiratory Infections in the Philippines.. PLoS One.

[72] Jartti T, Lee WM, Pappas T, Evans M, Lemanske RF (2008). Serial viral infections in infants with recurrent respiratory illnesses.. Eur Respir J.

[73] Olenec JP, Kim WK, Lee WM, Vang F, Pappas TE (2010). Weekly monitoring of children with asthma for infections and illness during common cold seasons.. J Allergy Clin Immunol.

[74] Rueckert RR, Pallansch MA (1981). Preparation and characterization of encephalomyocarditis (EMC) virus.. Methods Enzymol.

[75] Tate MD, Brooks AG, Reading PC (2008). The role of neutrophils in the upper and lower respiratory tract during influenza virus infection of mice.. Respir Res.

[76] Daley JM, Thomay AA, Connolly MD, Reichner JS, Albina JE (2008). Use of Ly6G-specific monoclonal antibody to deplete neutrophils in mice.. J Leukoc Biol.

